# Peanut leaf transcriptomic dynamics reveals insights into the acclimation response to elevated carbon dioxide under semiarid conditions

**DOI:** 10.3389/fpls.2024.1407574

**Published:** 2025-03-27

**Authors:** Haydee Laza, Bishwoyog Bhattarai, Venugopal Mendu, Mark D. Burow, Yves Emendack, Jacobo Sanchez, Aarti Gupta, Mostafa Abdelrahman, Lam-Son Phan Tran, David T. Tissue, Paxton Payton

**Affiliations:** ^1^ Department of Plant and Soil Science, Texas Tech University, Lubbock, TX, United States; ^2^ Division of Plant Science and Technology, University of Missouri-Columbia, Columbia, MO, United States; ^3^ Department of Agriculture, Agribusiness, and Environmental Sciences, Texas Agricultural and Mechanical (A&M) University-Kingsville, Kingsville, TX, United States; ^4^ Texas Agricultural and Mechanical (A&M) AgriLife Research, Texas Agricultural and Mechanical (A&M) University, Lubbock, TX, United States; ^5^ Cropping Systems Research Laboratory, United States Department of Agriculturertment, Agricultural Research Services (USDA-ARS), Lubbock, TX, United States; ^6^ Hawkesbury Institute for the Environment, Western Sydney University, Penrith, NSW, Australia; ^7^ Goanna Ag, Lubbock, TX, United States

**Keywords:** drought, water stress, gene expression, photorespiration, acclimation

## Abstract

**Introduction:**

Elevated atmospheric carbon dioxide [CO_2_] increases peanut carbon assimilation and productivity. However, the molecular basis of such responses is not well understood. We tested the hypothesis that maintaining high photosynthesis under long-term elevated [CO_2_] is associated with the shift in C metabolism gene expression regulation.

**Methods:**

We used a field CO_2_ enrichment system to examine the effects of elevated [CO_2_] (ambient + 250 ppm) across different soil water availability and plant developmental stages on the molecular responses in a peanut runner-type genotype. Plants under both [CO_2_] treatments were grown in semiarid conditions. We evaluated a comparative leaf transcriptomic profile across three periodic water deficit/re-hydration (well-watered/recovery) cycles throughout the growing season using RNAseq analysis.

**Results:**

Our results showed that the transcriptome responses were influenced by [CO_2_], water availability, and developmental stages. The traditional Mercator annotation analysis based on percentage total revealed that lipid metabolism, hormone biosynthesis, secondary metabolism, amino acid biosynthesis, and transport were the most regulated biological processes. However, our new approach based on the comparative relative percentage change per individual category across stages revealed new insights into the gene expression patterns of biological functional groups, highlighting the relevance of the C-related pathways regulated by elevated [CO_2_].

**Discussion:**

The photosynthesis analysis showed that 1) The light reaction was the most upregulated pathway by elevated [CO_2_] during water stress, 2) Photorespiration was downregulated across all stages, 3) Sucrose synthesis genes were upregulated by elevated [CO_2_] before stress, 4) Starch synthesis genes were upregulated by elevated [CO_2_] under drought periods, and 5) CO_2_ regulation of sucrose and starch degradation was critical under drought periods. Our findings provide valuable insights into the molecular basis underlying the photosynthetic acclimation response to elevated [CO_2_] in peanuts.

## Highlights

- The transcriptome responses were influenced by [CO_2_], water, and growth stages.
- The light reaction was the most up-regulated pathway by [CO_2_] during water stress.
- Sucrose synthesis genes were upregulated by [CO_2_] before stress.
- Starch synthesis genes were upregulated by [CO_2_] under drought periods.
- CO_2_ regulation of sucrose and starch degradation was critical under drought periods.
- Elevated [CO_2_] downregulated photorespiration across all stages.

## Introduction

Peanuts are primarily cultivated in semiarid regions and represent a significant source of protein and lipids for populations across the globe (Gangurde et al., 2019). Like most C3 plants, it is expected to benefit from rising atmospheric carbon dioxide concentration by enhanced photosynthesis and yield productivity under limited soil water availability Laza et al., 2021) and increasing the phytochemical shoot content, including total phenolics compounds (Novello et al., 2023). However, the positive CO_2_ effects may be diminished under extreme weather events, including high temperatures (Prasad et al., 2003). Wild and cultivated peanuts are unusual legumes because they have pod and seed development below ground, which may enhance carbon capture capability in future climates (Laza et al., 2021) (Gangurde et al., 2019).

Wild-type peanuts usually harbor a single genome (A or B). Thus, diploids (AA, BB) (Bertioli et al., 2011) and, rarely, tetraploids (AAAA, BBBB) can be found in wild natural populations. However, the peanut type suitable for production and human consumption, known as cultivated peanut (*Arachis hypogaea* L.), is an allotetraploid (AABB; 2n=40) (Lu and Pickersgill, 1993; Seijo et al., 2007; Bertioli et al., 2016), whose origin is from two diploid wild types (*Arachis ipaensis* and *Arachis japensis*) has been recently described (Bertioli et al., 2016). Although the increase in ploidy level can result in genetic gain, this ploidy event did not translate into significantly enhanced genetic diversity for peanut (Halward et al., 1991; Burow et al., 2001). Hence, efforts to increase peanut genetic background diversity, especially for seed productivity and disease-related traits, are among the main targets in peanut research. Peanut is an arid crop, and most likely impacted by projected stochastic environmental events such as intensified drought, heat waves, and rising atmospheric [CO_2_]. Hence, identifying key traits and their molecular basis will facilitate the development of climate-smart peanut varieties (Gangurde et al., 2019).

The impact of future climates on peanut productivity and leaf physiology has been previously studied (Vara Prasad et al., 1999; Prasad et al., 2003; Kottapalli et al., 2009; Laza et al., 2021). Like many plants, peanuts maintain homeostasis under different climatic scenarios through a coordinated pathways network. Thereby, carbon metabolism, including the coupling of establishes photosynthesis and respiration supplies a wide range of pathways with C-skeletons (Croteau et al., 2000). Three major photosynthesis-related pathways include light reactions, the Calvin cycle, and photorespiration. Photorespiration is a carbon C2 cycle that occurs when oxygen instead of CO_2_, binds to the Rubisco enzyme (Walker et al., 2016). Sucrose and starch are major components of carbohydrate (CHO) metabolism. The coordinated sugar dynamic is maintained by regulating of the hexose phosphate and triose phosphate pools. In general, we can get an idea of the sugar status by looking at the expression of “famine” and “feast” genes, which represent sugar depletion or abundance, respectively (Parrott et al., 2005) and play a pivotal role in transcriptomic reprogramming (Yokoyama et al., 2006). Although the transcriptomic analysis of peanuts grown under drought conditions has been previously studied (Jain et al., 2001; Dramé et al., 2007; Qin et al., 2011; Chen et al., 2013; Pruthvi et al., 2013; Chopra et al., 2014; Furlan et al., 2014), the interactive effect of elevated [CO_2_], high temperature and water deficit on peanut gene expression dynamics has not been addressed yet.

To gain a better understanding of the molecular mechanisms underlying the physiological, biochemical, and structural changes associated with elevated [CO_2_], we investigated the molecular basis of the photosynthetic acclimation response to elevated [CO_2_] throughout the life cycle as a function of plant growth stage and soil water availability. The present investigation was undertaken to answer the following questions: 1) Which biological processes are more responsive to elevated [CO_2_]? 2) Could the lack of leaf photosynthetic acclimation in peanuts be explained by the shift in gene expression caused by CO_2_ fertilization? and 3) Do peanut plants use different genes or expression levels to cope with subsequent water stress over time? Is this response altered by elevated CO_2_?

The acclimation response is usually explained by looking at the sink strength. In peanuts, developing new leaves and the symbiotic association with mycorrhizae and rhizobia might increase the sink strength, preventing the acclimation response from happening. The main objectives of this study were to 1) Identify relevant molecular mechanisms involved in the sustained enhanced photosynthetic rate induced by elevated [CO_2_], which prevent the acclimation response from happening, and 2) Examine if elevated [CO_2_] modifies. the capacity of peanut plants to acclimate to drought through transcriptomic reprogramming. We hypothesized that: 1) Enhanced photosynthetic rate at elevated [CO_2_] might be explained by the sustained-up regulation of genes involved in carbon metabolism; and 2) The photosynthetic acclimation response to elevated [CO_2_] might be altered in limited water conditions, due to differentially expressed genes induced by the interaction of these two factors compared to elevated CO_2_ alone.

## Materials and methods

### Plant material, growth conditions, and [CO_2_] treatments

This study (2015 and 2016) was conducted under semiarid conditions (water stress was not a studied treatment) in the research fields of the cropping system research laboratory at USDA-ARS Lubbock, Texas. *Arachis hypogaea* cv. C76-16; runner market type was planted at 2.5 cm depth at a planting density of 6 plants/m2 (60 x 103 plants ha^-1^) in 15 m row plots with 1.0 m row spacing. The experiment included two long-term [CO_2_] treatments (ambient; 400 ppm) and elevated; 650 ppm) under Canopy Evapotranspiration and Assimilation (CETA) chambers (Baker et al., 2014; Laza et al., 2021). Each treatment consisted of three replications. The chambers were placed on the plants in the field 10-15 days after sowing to ensure good stand establishment of selected areas. Peanut plants from both CO_2_ treatments were subjected to three water deficit stress and recovery cycles, and each water stress episode was evaluated by comparing three stages: well-watered stage before any stress, pre-water deficit (pwd), water deficit (wd), and well-watered recovery (ww rec). Details about experimental settings and chamber operations are described elsewhere (Laza et al., 2021, 2023).

### Transcriptome data collection

Although genomic analysis of peanuts has been previously conducted using a reference transcriptome, the completion of the peanut genome and the availability of the peanut reference genome (Bertioli et al., 2016), provide new opportunities to associate physiological responses to changes in transcription. In this study, we used a peanut reference transcriptome obtained from our sample profiling from leaves. We collected leaf samples at different growth stages from flowering to physiological maturity (R2-R8) through the growing season to develop the transcriptomic profile associated with the elevated [CO_2_] response. All samples were placed in liquid nitrogen immediately after collection and stored at -80°C for further analysis. Forty-two samples (42 leaves) were used for RNA extraction. For the acclimation response study, we used leaf samples collected at the three stages of the drought episode, including, pre-water deficit, water deficit, and recovery, 3 days following irrigation pre-water deficit (pwd), water deficit (wd) well-watered/recovery (ww-rec), 3 days following irrigation.

### RNA extraction, library preparation and sequencing

Frozen plant material (leaf) was ground using pre-cooled mortar and pestle with liquid nitrogen. Around 0.3 g (leaf) of the resulting fine powder was used to extract the total RNA using the RNeasy Sigma kit following the manufacturer’s protocol. Final elution was done with preheated (64°C) RNAse-free water. RNA quality and quantity were first monitored using the NanoDrop 2200 Spectrophotometer (Thermo-Scientific, Waltham, MA). Only samples with A260/230 > 1.9 and a concentration higher than 100 ng/µl were selected for further analysis. We checked the integrity of these samples using the tape station, looking for 28S and 18S bands. We conducted serial dilutions of the total RNA samples (using RNAse-free water as a solvent) based on the concentrations obtained from the Qubit™ 2.0 fluorometer (Invitrogen, Life Technologies, Grand Island, NY). We diluted to 100, 25, 15, and 7.5 ng/µl for cDNA library construction and quantification using the NeoPrep following the manufacturer protocol. The 42 cDNA constructed libraries were validated by checking the insert size using the Agilent tape station 2200. The final concentration was confirmed using the Qubit™ 2.0 fluorometer. The libraries were kept at -80°C, ready for sequencing. Samples were prepared according to Illumina’s RNA sequencing protocol and manufacturer’s instructions. The 42 cDNA libraries were divided into two groups of 24 and proceeded with two Rapid mode sequencing runs on the HiSeq 2500 instrument at the Center for Biotechnology and Genomics, Texas Tech University.

### Data analysis, functional annotation, and qRT-PCR analysis

Sequenced reads were obtained from bcl files using bcl2fastq software, and the quality of the Fastaq files was checked using FastQC software. The reads were then assembled into contigs to create a reference transcriptome using Trinity software. Reads were mapped to the reference transcriptome to determine differential expression using QSeq software. The list of DEGs was mapped onto biological pathways using MapMan software (Lohse et al., 2014).

The concentration of total RNA in each sample was determined with the MULTISKAN SkyHigh microplate spectrophotometer (Thermo Fisher Scientific Inc., USA). The concentration of total RNA for all samples was normalized to 1 μg μL-1 using RNase-free water. Subsequently, following the instructions provided, first-strand cDNA synthesis was performed using the QuantiTect reverse transcription kit (Qiagen, Hilden, Germany). All real-time qPCR (RT-qPCR) assays were performed on 96-well plates CFX96 Real-Time system (BIO-RAD, CA, USA). The reaction volumes were 10 μL and contained 5 μL Sso Advanced Universal SYBR Green Supermix (BIO-RAD, CA, USA), three μL cDNA templates, and two μL primer pair mix (10 pmol each primer). In each run, triplicates of no-template control (NTC) comprising DEPC-treated water were incorporated. The thermal cycling conditions consisted of 40 cycles, starting with an initial denaturation at 96°C for 10 seconds, then annealing at 60°C for 30 seconds, and an extension phase at 72°C for 30 seconds. Melting curves were obtained using a thermal melting profile performed after the last PCR cycle: 65°C for 10 seconds, followed by a constant increase in the temperature between 65°C and 95°C. All the specific primer pairs employed in this study are given in **Supplementary Table S1**. Amplicon-based fluorescence thresholds were used to obtain the CT values. The eukaryotic elongation factor 1 beta (ELF1B) gene was used as a reference gene in the RT-qPCR analysis of RNA samples from three biological replicates (n = 3 × 2 technical replicates).

### Statistical analysis

We performed the leaf transcriptomic analysis to elucidate the mechanistic basis of our physiological findings (Laza et al., 2021). To better understand the underlying mechanisms of the acclimation response, we conducted eight vertical pair comparisons between the [CO_2_] treatments and three horizontal pair comparisons between growth stages (C8-R2/R3, C9-R3/R4, and C10-R2/R4) and soil water availability periods (pwd, wd, ww-rec) for each [CO_2_] treatment (ambient, 400 ppm and elevated, 650 ppm) independently (**Table 1**). Data was expressed for each time point as the percentage of total DEGs and as the percentage change (i.e the change in DEGs as the percentage of the baseline between growth stages). Here, we focused our analysis on the effect of elevated [CO_2_] on 1) the expression genes per functional of group across growth stages to examine if the lack of leaf photosynthetic acclimation in peanuts (Laza et al., 2021) could be explained by the shift in gene expression caused by CO_2_ fertilization in one or more pathways and 2) plant ability to cope with subsequent water stress over time. The qualitative gene expression (up/down) was analyzed for each functional group (encoding gene transcripts) by examining the regulation type across stages for each DEG. This analysis provided new insights regarding the expression dynamics. DEGs were defined as genes with at least |log2 fold-change (FC)|>1 and adjusted P-value ≤ 0.05. T test (at *P<0.05*) was conducted to determine differences between the number of DEG between stages.

**Table 1:** Summary of the 8-time points sample collection. Leaf samples of peanut plants grown under ambient atmospheric [CO_2_] (400 ppm) and elevated [CO_2_] treatments were collected to perform the comparison (C) analysis across different reproductive developmental stages (R) and soil water availability. C1 (beginning peg development-R2 at well-watered/pre-water deficit-pwd), C2 (beginning pod-development-R3 at the first water deficit-wd1), C3 (full pod-R4 at the first well-watered/recovery-rec1), C4 (beginning seed-R5 at the second water deficit-wd2), C5 (full seed-R6 at the second well-watered/recovery-rec2 ), C6-C7 (beginning maturity-R7 at third water deficit-wd3, followed by the third well-watered/recovery-rec3), and C8 (harvest maturity-R8, well-watered).

Comparisons	C1	C2	C3	C4	C5	C6	C7	C8

Soil water content	pwd	wd1	rec1	wd2	rec2	wd3	rec3	Harvest
Plant growth stage	R2	R3	R4	R5	R6	R7	R7	R8
	C9 C10							
	C11							

Each plot represents three biological replications.

## Results

### Summary of the transcriptomic analysis

**Figure 1:**
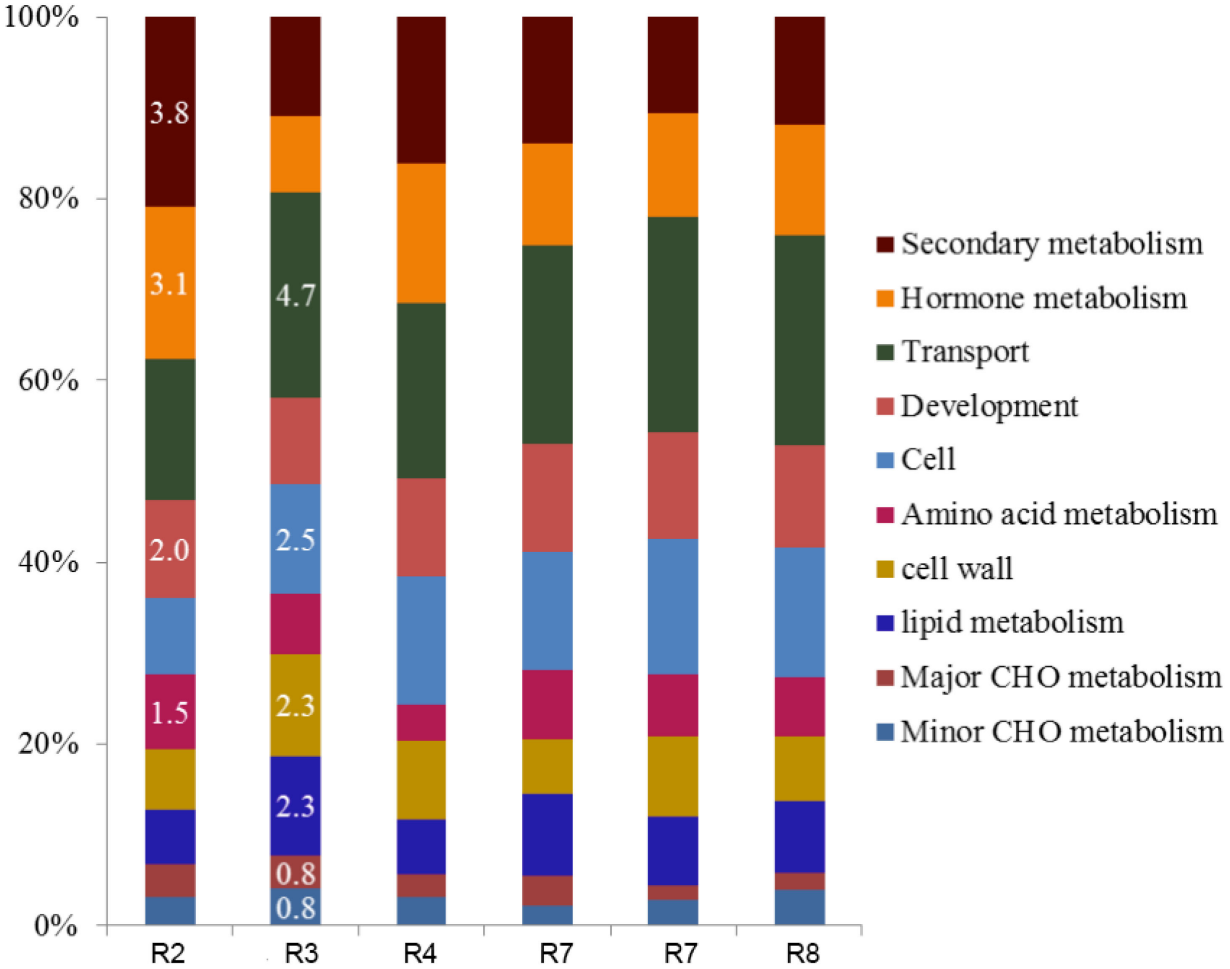
Effect of elevated CO_2_ on the percentage of total (%) differentially expressed genes (DEGs) per functional group resulted from the peanut leaf transcriptomic. Differential gene expressions as a percentage of the total DGEs in six selected data points, representing different plant growth stages with different soil water availability: pre-water deficit (R2), first water deficit (R3), first recovery (R4), third water deficit (R7), third recovery (R7), and physiological maturity-harvest (R8). Carbohydrate-CHO.

Overall, the number of DEGs increased over time, with a significant peak from pre-water deficit (R2; 1182) to first recovery (R4; 3044) (**Table 2**). The leaf transcriptomic analysis across different growth stages revealed a similar gene expression pattern in response to water deficit and recovery. Thus, 34 out of 35 biological processes (identified by Mercator software) were induced in peanut leaves, regardless of the atmospheric [CO_2_] (**Figure 1**). Furthermore, for the seven data points considered, the differential expression was more pronounced for protein, RNA, and signaling categories, with maximum values up to 9.4%, 6.3%, and 5.3%, respectively. Conversely, an increased number of group functions were less responsive to CO_2_. These functional groups include minor carbohydrate (CHO) metabolism, polyamine metabolism, biodegradation of xenobiotics, redox, co-factor/vitamin metabolism, and TCA (tricarboxylic acid) pathways, showing less than 0.2% percent of the total differentially regulated responses. Moreover, gluconeogenesis, oxidative pentose phosphate (OPP), C1-metabolism, tetrapyrrole synthesis, S assimilation, and N metabolism were not induced by elevated [CO_2_] at the early plant reproductive stage before any water deficit event.

**Table 2:** Summary of differentially expressed genes (DEGs) induced by elevated [CO_2_] in peanut leaves.

	Pre-water deficit (R2)	First water deficit (R3)	First recovery (R4)
No. DEGs	1182	2979	3044
No. Assigned	678	1469	1845
Unknown	628	1331	1729
No. Ontology	50	138	116
Function groups	504	1510	1199
Down-regulated	655	1197	1151
Up-regulated	527	1782	1893
% Down-regulated	45	40	38
% Up-regulated	55	60	62

Changes across different developmental stages (R2, R3, and R4) and soil water availability (pre-water deficit, water deficit, and well-watered recovery) are presented.

### Expression of biological processes and patterns under elevated [CO_2_]

The number of categories per functional group varied with growth stage/soil water availability and exhibited different patterns while transitioning from one stage to the other (**Table 3**). Although a higher number of functional groups (3044) was found during the first re-hydration event (Rec-1), the dynamic of quantified expression (number of functional groups) was different for each biological process. To better understand the effect of elevated [CO_2_] on gene expression dynamics, we looked at 1) each time point (ambient control *vs*. elevated [CO_2_]) and 2) between time points (each [CO_2_] treatment individually at different stages (well-watered[ww] *vs* water deficit [wd] periods). The quantitative gene expression pattern (number of functional groups differentially expressed between the treatments) for each time point was classified as 1a) modified (when the number of functional groups between treatments was different), or 1b) unchanged (same number of categories). This response also changed across growth stages. Thus, four major quantitative gene expression patterns were identified: 2a) up-pattern (the number of categories increases from one stage to the other); 2b) down-pattern (the number of categories is reduced from one stage to the other); 2c) unchanged-pattern (the number of categories remains the same), and 2d) mixed-pattern (were the response from the first transition between stages changed in the next transition).

**Table 3:** Percentage change of functional groups differentially expressed across growth stages (R2-R4) with different soil water availability (pre-water deficit [pwd] at R2, water deficit[wd] at R3, and well-watered recovery [ww/rec] at R4).

Functional group	pwd R2	wd R3	ww R4	% change pwd/wd	Pattern Type	% change wd/ww	Pattern Type	% change pwd/ww	Pattern Type
photosynthesis	3	19	16	533	up	-16	down	433	up
major CHO	6	26	8	333	up	-69	down	33	up
minor CHO	2	24	11	1100	up	-54	down	450	up
glycolysis	2	5	6	150	up	20	up	200	up
fermentation	3	4	1	33	up	-75	down	-67	down
gluconeogenesis	0	1	1	na	up	0	nc	na	up
OPP	0	1	2	na	up	100	up	na	up
tricarboxylic acid	1	6	1	500	up	-83	down	0	nc
mitochondrial electron transport	2	4	7	100	up	75	up	250	up
cell wall	11	74	39	573	up	-47	down	255	up
lipid metabolism	9	63	17	600	up	-73	down	89	up
N-metabolism	0	2	3	na	up	50	up	na	up
amino acid metabolism	15	28	17	87	up	-39	down	13	up
S-assimilation	0	0	1	na	up	na	up	na	up
metal handling	6	9	3	50	up	-67	down	-50	down
secondary metabolism	33	57	50	73	up	-12	down	52	up
hormone metabolism	26	39	41	50	up	5	up	58	up
Co-factor, vitamin	6	7	9	17	up	29	up	50	up
tetrapyrrole synthesis	2	1	8	-50	down	700	up	300	up
stress biotic	32	95	102	197	up	7	up	219	up
redox	1	22	17	2100	up	-23	down	1600	up
polyamine metabolism	4	3	3	-25	down	0	nc	-25	down
nucleotide metabolism	5	15	8	200	up	-47	down	60	up
biodegradation of xenobiotics	3	7	3	133	up	-57	down	0	nc
C1-metabolism	0	1	1	na	up	0	nc	na	up
miscellaneous	56	169	82	202	up	-51	down	46	up
RNA	58	165	155	184	up	-6	down	167	up
DNA	7	49	41	600	up	-16	down	486	up
protein	84	205	218	144	up	6	up	160	up
signaling	49	139	151	184	up	9	up	208	up
cell	20	70	52	250	up	-26	down	160	up
development	24	61	39	154	up	-36	down	63	up
transport	34	140	86	312	up	-39	down	153	up
Not assigned	678	1469	1845	117	up	26	up	172	up
Total	1182	2980	3044						nc
% mean				307		6		191	

Data for the percentage change between the pwd and wd (R2 to R3), wd and ww/Rec (R3 to R4), pwd and rec (R2-R4) are presented. Each data point represents an average between three biological replications. “nc” represents no change, and “na”, no available/calculated. Carbohydrate-CHO, Oxidase Pentose Phosphate-OPP.

The mixed pattern includes a variety of combinations resulting from the induction, inhibition, or unchanged expressions, such as up/down (increased number of DEGs from pre-water deficit (R2) to 1st water deficit (R3), followed by a reduction from 1st water deficit (R3) to 1st recovery (R4), down/up (the opposite effect described for up/down), unchanged/up, unchanged/down, etc. Understanding the gene expression dynamics is important to characterizing the molecular basis accurately and determining which factors (environmental, developmental, or a combination of both) drive the transcriptomic reprogramming over time. It also helps elucidate whether the responses are associated with current stress/signal or memory stress (prior stress within the organism’s life span or even transgenerational background).

**Figure 2:**
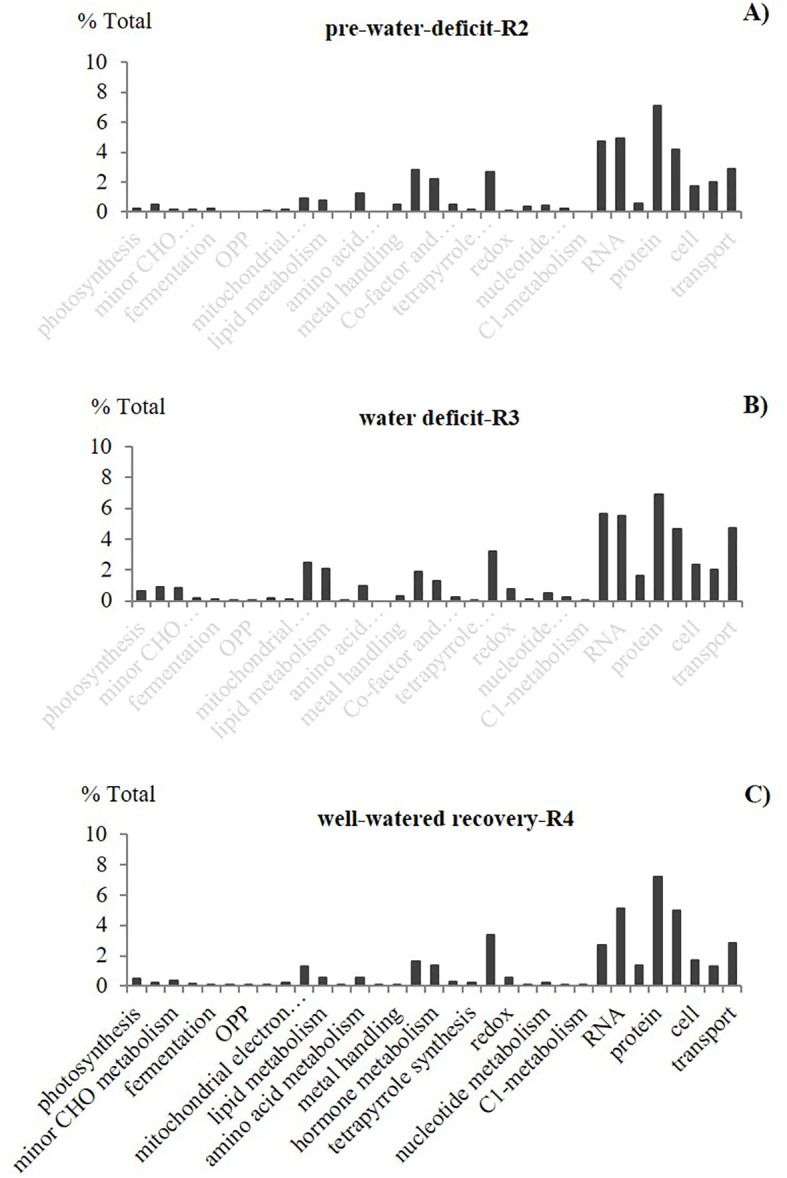
Effect of elevated CO_2_ on the percentage of total differentially expressed genes (DEGs) for different functional groups. Leaf transcriptomic profile from pair vertical comparisons between the two CO_2_ treatments (control, ambient 400 ppm and elevated 60 ppm) at selected growth stages with different soil water availability: **(A)** pre-water deficit (R2), **(B)** first water deficit (R3), and **(C)** well-watered/first recovery (R4)], using the “percentage of total” approach (% indicates the effect of elevated CO_2_ over ambient treatment). Carbohydrate-CHO, the oxidative pentose phosphate-OPP. Stats criteria for differentially expressed genes. Genes with |log2 fold-change|>1and adjusted *P-value uc1≤ .05* (FDR (false discovery rate).

In our study, we used two approaches for the gene expression analysis. Our results showed different expression patterns for the two approaches. The first approach, “Percentage of total” (based on the percentage of the total DEGs per function), showed that the expression trend and dynamic were very similar in all the data points, regardless of the growth stage and water availability (**Figure 2**). This type of analysis, usually presented as a pie graph of functions (software generated), is perhaps one of the most popular approaches currently used for RNA data interpretation. However, because this functional analysis is based on the number of different types of transcripts per biological function, it doesn’t represent normalized values; therefore, functions requiring the greater number of genes are more likely to have greater percentage of total than functions requiring a low number of genes. Hence, the % of Total analysis doesn’t represent an actual expression pattern since functions and pathways differ on the number of genes/Transcription Factors (TF) required.

**Figure 3:**
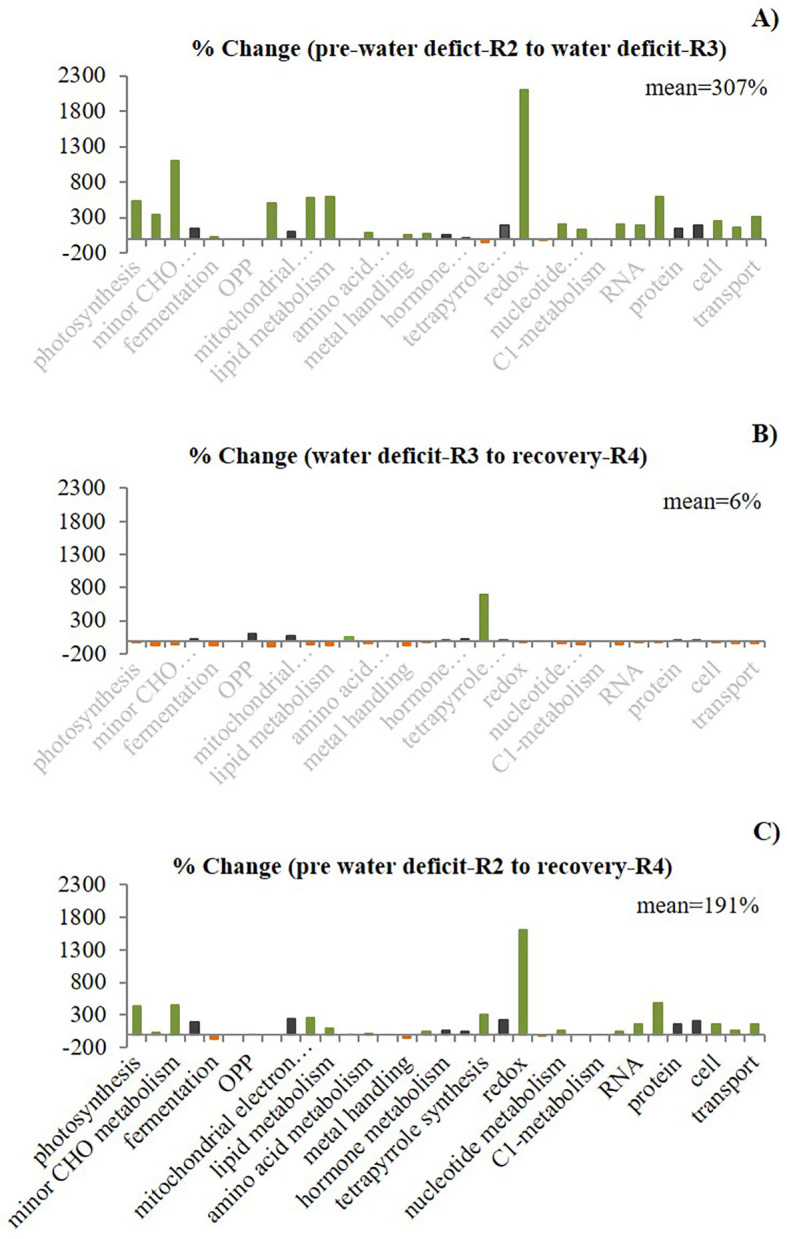
Effect of elevated CO_2_ on the percentage change of differentially expressed genes (DEGs) per functional group and between stages (first stage as a baseline) in field-grown peanuts. The gene expression regulation exhibited conserved and altered patterns represented by three colors. Black indicates similar pattern (“no change pattern”) for the three comparisons between stages **(A)** R2/R3, **(B)** R3/R4 and **(C)** R2/R4). for the three comparisons), green color indicates an increase (“up pattern”) in the percentage change. Orange indicates a decrease in the percentage change. The % change mean across all the functional groups for each comparison between stages showed distinct expression patterns.

The second approach, “Percentage Change,” revealed different gene expression patterns than the percentage of the total approach. Here, we demonstrated that using the percentage change (defined as the change in DEGs as a percentage of the baseline for a given function between two stages) while transitioning across stages provides a better representation of expression dynamics, with more accurate identification of relevant functions which have been altered between treatments. Thus, this analysis revealed distinct expression patterns for each transition (**Figure 3**), with the highest percentage change (mean of 307% averaged across all functions) during the first transition from high to low soil water content. This %mean of differential expression was reduced to almost zero (6%) when plants transitioned from severe water deficit to the following recovery period, and the change in the %mean between the two well-watered periods (pwd and recovery) was also high (191%). In contrast with the “Percentage of total” approach, we were also able to identify the most relevant biological functions associated with elevated [CO_2_] and water deficit using the “Percentage change” approach. The results showed that C metabolism-related functions were among those with the highest percentage change, suggesting that although the percentage of the total was low for these functions, the relative abundance of transcripts was significantly altered from the baseline stage. Therefore, we selected these functions for further analysis.

### Photosynthesis pathways regulation under elevated [CO_2_]

**Figure 4:**
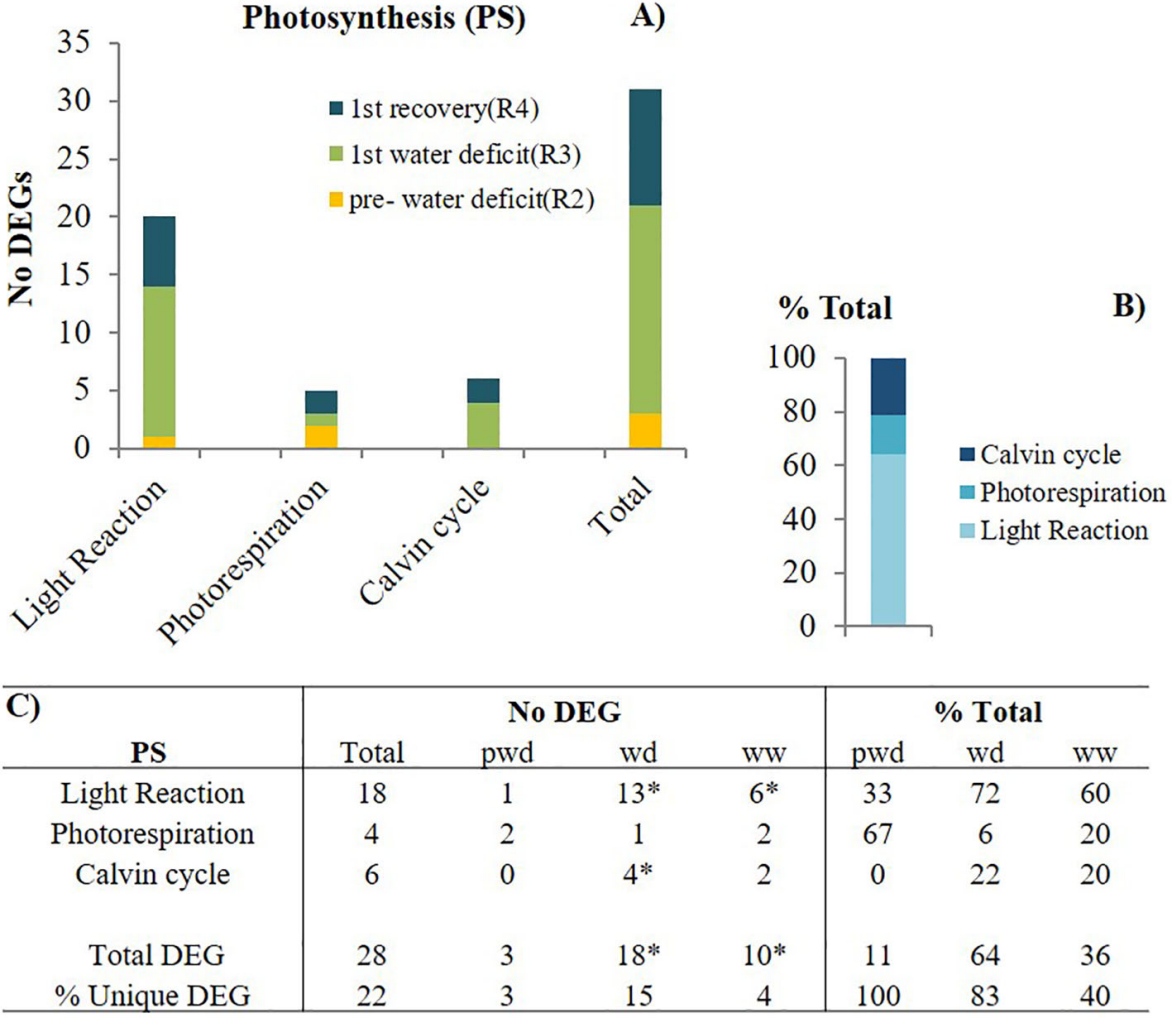
Photosynthetic (PS)-related pathways modulation by elevated [CO_2_]. **(A)** the number (No) of differentially expressed genes (DEGs) per pathway during pre-water deficit (at the R2 developmental stage), first water deficit (R3), and first recovery (R4); **(B)** the percentage of total for each pathway, averaging across stages; and **(C)** the summary of total and unique DEGs per stage and pathway. Stats criteria for determining the DEGs was *P-value uc1≤ .05* and |log2 fold-change|>1. The * indicates significant difference at *P*<0.05, T test (pwd as control).

We examined the gene expression of the C-related pathways, including photosynthesis (PS), carbohydrate (CHO) metabolisms, and respiration. First, we pooled all the PS-DEG, further subdivided into pathways: 1) light reactions/photosynthetic electron transport chain, 2) C3-Calvin cycle, and 3) C2 cycle- photorespiration. Across the first stress cycle (pre-water deficit (R2), 1^st^ water deficit (R3), recovery (R4), and the following recovery, the greater number of unique DEGs (18) were associated with the light reaction. In contrast, the C3 and C2 cycles were less impacted, with only 6 and 4 unique DEG, respectively (**Figure 4**). We observed that elevated [CO_2_] upregulated one of the photosystem II genes (mutarase; chlorophyll-binding to D1) in the light reactions (8.7 log2FC), coupling with a downregulation of the photorespiratory pathway. (-7.6 log2FC) (**Supplementary Table S2**). During the first recovery, a significant downregulation of two genes encoding a reduction phase enzyme, Glyceraldehyde-3-phosphate dehydrogenase (GAPDH), and a regeneration phase enzyme, Fructose 1,6-bisphosphatase (FBAse), with a greater reduction (-11.8 log2FC).

### Carbohydrate metabolism pathways regulation under elevated [CO_2_]

**Figure 5:**
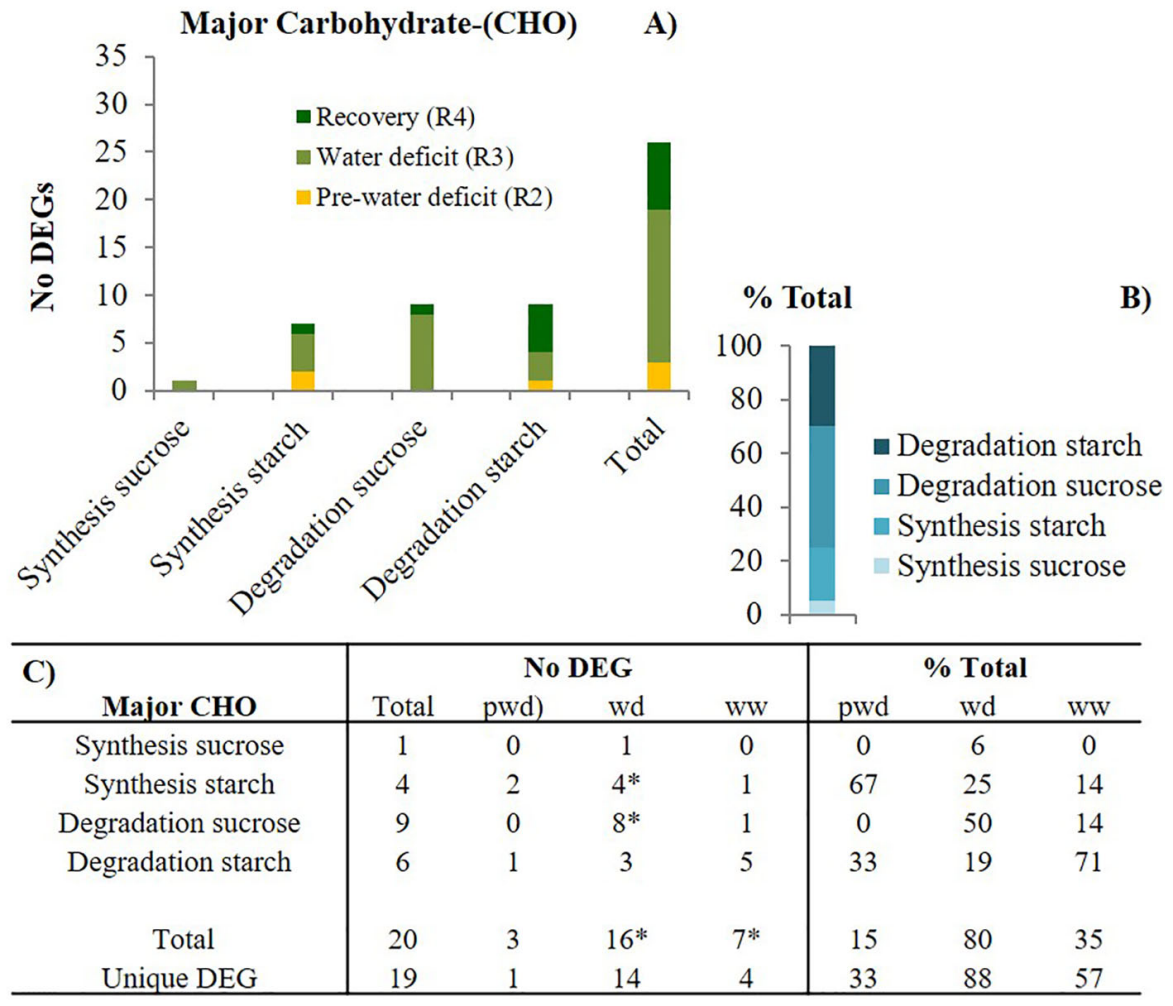
Major carbohydrate (CHO) metabolism-related pathways modulation by elevated [CO_2_]. **(A)** the number of differentially expressed genes (DEGs) per pathway during pre-water deficit (at the R2 developmental stage), first water deficit (R3), and the first recovery (R4); **(B)** the percentage of total for each pathway, averaging across stages; and **(C)** summary of total and unique DEGs per stage and pathway. Stats criteria for determining the DEGs was P-value uc1≤ .05 and |log2 fold-change|>1. The * indicates significant difference at *P*<0.05, T test (pwd as control).

We further investigated three relevant carbohydrate (CHO) metabolism pathways, including the major-CHO and minor-CHO. The major-CHO metabolism includes synthesis and degradation of sucrose and starch. The major-CHO metabolism was controlled by 20 DEGs across three stages (pwd-R2, wd1-R3, and ww1-R4), from which 19 were unique and stage-specific (**Figure 5**). Like photosynthesis, the number of DEGs significantly increased from pre-water deficit (3) to first water deficit (16). The results showed that elevated [CO_2_]: 1) upregulated the starch degradation genes including the starch cleavage alpha-amylase (AMY1, 3.2 log2FC) before the water stress (pwd) (**Supplementary Table S3**), 2) upregulate the sucrose synthesis (FBPase, 1.78 Log_2_FC), starch synthesis, (ADP-glucose pyrophosphorylase-(AGPase, 1.00 log2FC, and starch synthase 1.61 log2FC) and starch branching (6.84 log2FC) during the water deficit stress (wd) period, and 3) induced both upregulation of starch synthesis(AGPase, 5.6 log2FC) and starch degradation (AMY1, 2.2 log2FC), as well as enhanced sucrose degradation (vacuolar invertase, 1.3 log2FC) during the well-watered recovery phase (Rec).

### Respiratory pathways regulation under elevated [CO_2_]

Our results showed that, in general, transcripts related to glycolysis, TCA, and Electron Transport Chain (ETC) were upregulated, indicating that C use was enhanced by elevated CO_2_ in peanuts. Similarly, to PS and major CHO, the trend for respiratory pathways was the lower number of functional groups before any water deficit compared to the first water deficit and the first recovery periods. During the pre-water deficit, the TCA pathway was not significantly affected, with aconitase as the only TCA enzyme differentially regulated. This enzyme was significantly inhibited (-7.8 log2FC) (**Supplementary Table S4**) by elevated [CO_2_] before the water deficit period. Furthermore, most of the mitochondrial genes were not altered, but ATP synthesis was significantly reduced (-1.3 log2FC). Similarly, metabolite transport function declined (-1.7 log2FC) in the mitochondrial. Although during pre-water deficit, glycolysis-related transcripts were upregulated, the major respiratory pathways, such as TCA, and ETC, were inhibited. A mix of up/down functions might be used for smaller adjustments to keep the C metabolism balanced. Conversely, when plants were exposed to severe water deficit conditions, the number of functional groups with DEGs significantly increased. Moreover, pathways not induced by elevated [CO_2_] during well-watered conditions, including gluconeogenesis and oxidative pentose phosphate, were induced during severe water stress periods. Likewise, most of the glycolysis, TCA, and ETC-related transcripts were upregulated, suggesting that when the peanut plants experienced low soil water content, respiration was enhanced.

## Discussion

### Enhanced light reactions and Calvin cycle by elevated [CO_2_] during water stress

Reductions in photosynthesis at elevated [CO_2_] have been associated with lower leaf N, protein, and Rubisco content (Moore et al., 1999) and reduced ETC or Rubisco activase (RCA) content/activity (Sage et al., 2008), among other factors. Previous studies confirmed that peanut (runner type) protein and Rubisco content were significantly reduced when grown in enriched CO_2_ (720 ppm, daytime) (Vu and Allen, 2009). High temperature has been found to reduce the light activation of Rubisco by RCA in several C3 plant species, including cotton and wheat, when the atmospheric temperature reaches values higher than 35°C and 30°C, respectively (Feller et al., 1998). In our gene expression study, we found that the Rubisco active form was downregulated. Our results suggest that high photosynthesis at elevated [CO_2_] was mostly driven by the upregulation of the photosynthetic ETC and Calvin cycle and, to a lesser extent, the inhibition of RCA. In this context, previous studies found that the reduction in RCA content must be significant (-55%) to impact Rubisco activation (Yamori and von Caemmerer, 2009). It is well documented that Rubisco activation requires the incorporation of CO_2_ and Mg^+2^ (Buchanan et al., 2015), but the mechanism of light activation by RCA is yet unknown. Hence, we could not identify the causative factors of RCA downregulation in this study, which needs further investigation.

We can assume that enhanced related photosynthetic pathways, such as light reactions and the Calvin cycle, could lead to enhanced sugar production. Depending on the coupling between C fixation and utilization, this response could result in carbohydrate accumulation or enhanced growth. In this context, sugar accumulation has been identified as a negative feedback mechanism of photosynthetic acclimation to elevated [CO_2_] (Sims et al., 1998). Other studies showed that the magnitude and type of carbohydrate accumulation might also vary with N availability (Aranjuelo et al., 2013; Noguchi et al., 2015). Thus, studies integrating C and N metabolism will increase our understanding of the underlying basis of the acclimation response under elevated [CO_2_].

Data from the first recovery showed significant downregulation of two genes encoding a reduction phase enzyme, Glyceraldehyde-3-phosphate dehydrogenase (GAPDH) and a regeneration phase enzyme, Fructose 1,6-bisphosphatase (FBAse), GAPDH catalyzes the interconversion between 1, 3-Bisphospho-glycerate and Glyceraldehyde 3-phosphate (GAP). At the same time, FBAse converts Fructose 1, 6-biphosphate into Fructose 6 phosphate by inorganic P removal. The greater inhibition of FBAse may be due to its involvement in several related pathways (Noguchi et al., 2015). The downregulation of these enzymes might suppress the Calvin cycle and, thereby generate less sugar production. If so, this inhibition does not reflect the enhanced photosynthetic rate observed at this stage and cannot be used as a proposed molecular basis of this physiological trait.

### Enhanced sucrose synthesis by elevated [CO_2_] during water stress

Our results showed that the major CHO metabolism-related genes followed a similar pattern as the PS-related genes, with the number of DEGs significantly increased in the transition from pre-water deficit to first water deficit stress, suggesting that drought enhanced the CHO gene expression under elevated [CO_2_]. Moreover, sucrose metabolism-related transcripts were not affected during optimal soil water conditions. Before any water stress, C metabolism was exclusively controlled by starch metabolism, with 67% and 33% of the total DEGs involved with synthesis and degradation, respectively. In contrast, when plants experienced severe soil water deficit, C metabolism was controlled by both sucrose and starch metabolism. The significantly reduced number of DEGs during the first recovery event suggests that CHO metabolism-related pathways are more responsive to the interactive effect of water deficit and drought than to elevated [CO_2_] alone. Enhanced sucrose synthesis was only found during the water deficit period. Sucrose can be synthesized via sucrose synthase and sucrose-phosphatase synthase (Baschetti, 1997). Its synthesis is usually activated with the accumulation of hexose phosphate (Guy et al., 1992). Thus, the increased sucrose synthesis in the enriched CO_2_ environment might be associated with sugar accumulation and subsequent activation of the sucrose-phosphate enzyme.

Conversely, our results showed that the regulation of starch metabolism seems to take place during both low and high soil water availability. Across different stages (pre-water deficit; R2, first water deficit; R3, and first recovery; R4), the starch metabolism was similarly controlled by the upregulation of two common enzymes: 1) ADP-glucose pryrophoshorylase for starch synthesis, and 2) Alpha-amylase for starch degradation. Starch synthesis related-transcripts were enhanced at elevated [CO_2_] and regulated via ADP-glucose pyrophosphorylase or Granule-bound starch synthase. In contrast, other unique transcripts, which were stage-specific, were used to generate distinct molecular strategies to cope with different scenarios. Here, during high soil water content periods, elevated [CO_2_] increased starch synthesis by the upregulation of genes encoding ADP-glucose pryrophoshorylase enzyme only, which is further regulated by the ratio of inorganic P and the photosynthetic intermediate, 3-phosphogycerate (3-PGA) (Ballicora et al., 2004).

We found that induced differential gene expression by elevated [CO_2_] was more pronounced for protein, RNA, and signaling categories. This suggests that many genes associated with these processes are responsive to atmospheric [CO_2_] changes and remain differentially regulated throughout the entire plant life cycle. Similarly, the transcriptomic analysis showed that stress, RNA, miscellaneous, protein, and signaling were among the functional categories with the most responsive genes to drought and high temperature in poplar species (Jia et al., 2017).

Most of the carbohydrate metabolism-related transcripts were upregulated, suggesting that the enhanced C assimilation rate at elevated [CO_2_] in peanuts can be explained by an increased sugar metabolism. Furthermore, our gene expression analysis confirmed increased leaf photosynthesis (*A_net_*) at elevated [CO_2_] in peanuts, previously documented in (Laza et al., 2021).

Increased *A_net_* could be the result of 1) upregulation of photosynthetic-related pathways such as the light reactions and Calvin cycle pathway or as an indirect inhibition of respiration and photorespiration processes, leading to an overall increased *A_net_*, with similar gross photosynthetic rate. As expected, higher atmospheric [CO_2_] reduced photorespiration, which might explain the enhanced *A_net_* at the early reproductive stage (R2) and optimal soil water availability in enriched CO_2_ peanut agroecosystems. In agreement with our results, photorespiration inhibition with increasing atmospheric [CO_2_] has been reported in many plant species (Long et al., 2004).

Interestingly, transcripts related to the Calvin cycle were not affected by elevated [CO_2_] (zero DEG) until plants experienced severe water deficit conditions. These results indicate that the previously reported enhanced leaf *A_net_* during pre-water deficit (Laza et al., 2021) cannot be directly explained by changes in the Calvin cycle pathway. Instead, regulating other C-related processes could be responsible for this response. We also found that elevated [CO_2_] upregulated one of the photosystem II genes (mutarase; chlorophyll-binding to D1) in the light reactions and downregulated the photorespiratory pathway. Walker et al. (2016) found that in the absence of stress (abiotic or biotic), photosynthesis is most likely to increase by blocking the C2 cycle/photorespiratory pathway. In agreement with our findings, photorespiration was also significantly inhibited in *Arabidopsis* plants grown at high atmospheric [CO_2_] (Zinta et al., 2014). Although photorespiration has been considered an inefficient process attached to photosynthesis, part of the C lost through this mechanism can be recovered and has been quantified (24-38%) for other C3 crops like rice and soybean (Busch et al., 2013).

### High C assimilation under CO_2_ is regulated by a different set of genes across stages

To understand the molecular basis of the photosynthetic acclimation response to elevated [CO_2_] in peanuts under semiarid conditions, we examined the gene expression of relevant C-related pathways. Our gene expression analysis showed that very few common DEGs were found across stages. This suggests that different regulatory checkpoints delivered the same physiological outcome (the enhanced photosynthetic rate at elevated [CO_2_]). In this context, we identified two molecular strategies used by peanuts under elevated [CO_2_] before and during water deficit stress.

**Strategy-1**: Before the water stress, the downregulation of photorespiration was the major regulatory mechanism, but elevated [CO_2_] did not induce the Calvin cycle-related transcripts. However, elevated [CO_2_] down-regulated photorespiratory genes and upregulated the 3-PGA during the pre-water deficit period. This relative abundance of 3-PGA at elevated [CO_2_] compared to ambient could have triggered the observed ADP-glucose pyrophosphorylase activation. The enhanced photosynthetic rate was not associated with changes in the C3 reductive photosynthetic carbon cycle (Calvin cycle; zero DEG). Similarly, in Arabidopsis, an increased C assimilation was not explained by changes in Calvin cycle metabolites (Noguchi et al., 2015).

**Strategy-2**: During water stress, at a later reproductive stage, the molecular mechanism responsible for the enhanced photosynthetic rate had more genes involved in the photosynthetic electron transport chain. This regulation might be related to an increased light absorption capacity, reflected in the greater number of DEG related to light harvesting complex (LHC). An increased light absorption capacity might help speed up the electron transport and C fixation. Similarly, an increased abundance of transcripts related to the Calvin cycle and upregulation of sucrose synthesis suggests the most likely molecular strategy responsible for the enhanced net C assimilation in enriched [CO_2_] systems during severe water deficit periods.

## Concluding remarks

This study reveals the complex dynamics of the peanut leaf transcriptome in response to the combined effect of plant development, soil water availability, and atmospheric [CO_2_]. Based on quantitative and qualitative gene expression analysis, the molecular differential responses were more pronounced during water deficit periods than during periods of sufficient water. Our research suggests that high C assimilation under elevated CO_2_ is associated with the significantly altered expression of transcripts that regulate C metabolism pathways, including the downregulation of transcripts controlling photorespiration. Our findings provide valuable insights into the molecular basis underlying the photosynthetic acclimation response to elevated [CO_2_] in peanuts.

## Data Availability

The original contributions presented in the study are publicly available. This data can be found here: https://www.ebi.ac.uk/biostudies/arrayexpress, accession number E-MTAB-14869 and https://dx.doi.org/10.3389/fpls.2024.1407574.

## Supplementary material

Supplementary Table 1 List of genes and primers used for Real Time-Polymerase Chain Reaction (RT-PCR) gene expression validation of RNAseq transcriptomic in peanuts under elevated [CO_2_] and water stress.

Supplementary Table 2 Leaf RNAseq analysis summary-Photosynthesis.

Supplementary Table 3 Leaf RNAseq analysis summary-CHO.

Supplementary Table 4 Leaf RNAseq analysis summary-Respiration.

## References

[B1] Aranjuelo I. Sanz-Sáez Á. Jauregui I. Irigoyen J. J. Araus J. L. Sánchez-Díaz M.. (2013). Harvest index, a parameter conditioning responsiveness of wheat plants to elevated CO2. J. Exp. Bot. 64, 1879–1892. doi: 10.1093/jxb/ert081 23564953 PMC3638836

[B2] Baker J. T. Gitz D. C. Payton P. Broughton K. J. Bange M. P. Lascano R. J. (2014). Carbon dioxide control in an open system that measures canopy gas exchanges. Agron. J. 106, 789–792. doi: 10.2134/agronj13.0450

[B3] Ballicora M. A. Iglesias A. A. Preiss J. (2004). ADP-glucose pyrophosphorylase: A regulatory enzyme for plant starch synthesis. Photosynth. Res 79, 1–24. doi: 10.1023/B:PRES.0000011916.67519.58 16228397

[B4] Baschetti R. (1997). Sucrose metabolism. N. Z. Med. J 110, 43. doi: 10.1002/9780470015902.a0021259 9066573

[B5] Bertioli D. J. Cannon S. B. Froenicke L. Huang G. Farmer A. D. Cannon E. K. S.. (2016). The genome sequences of Arachis duranensis and Arachis ipaensis, the diploid ancestors of cultivated peanut. Nat. Genet. 48, 438–446. doi: 10.1038/ng.3517 26901068

[B6] Bertioli D. J. Seijo G. Freitas F. O. Valls J. F. M. Leal-Bertioli S. C. M. Moretzsohn M. C. (2011). An overview of peanut and its wild relatives. Plant Genet. Resour. 9, 134–149. doi: 10.1017/S1479262110000444

[B7] Buchanan B. B. Gruissem W. Jones R. L. (2015). Biochemistry & Molecular biology of plants. Biochem. Mol. Biol. Plants 1264.

[B8] Burow M. D. Simpson C. E. Starr J. L. Paterson A. H. (2001). Transmission genetics of chromatin from a synthetic amphidiploid to cultivated peanut (Arachis hypogaea L.): Broadening the gene pool of a monophyletic polyploid species. Genetics 159, 823–837. doi: 10.1093/genetics/159.2.823 11606556 PMC1461827

[B9] Busch F. A. Sage T. L. Cousins A. B. Sage R. F. (2013). C3 plants enhance rates of photosynthesis by reassimilating photorespired and respired CO2. Plant Cell Environ. 36, 200–212. doi: 10.1111/j.1365-3040.2012.02567.x 22734462

[B10] Chen X. Zhu W. Azam S. Li H. Zhu F. Li H.. (2013). Deep sequencing analysis of the transcriptomes of peanut aerial and subterranean young pods identifies candidate genes related to early embryo abortion. Plant Biotechnol. J. 11, 115–127. doi: 10.1111/pbi.12018 23130888

[B11] Chopra R. Burow G. Farmer A. Mudge J. Simpson C. E. Burow M. D. (2014). Comparisons of *de novo* transcriptome assemblers in diploid and polyploid species using peanut (Arachis spp.) RNA-Seq data. PloS One 9. doi: 10.1371/journal.pone.0115055 PMC4281230 25551607

[B12] Croteau R. Kutchan T. M. Lewis N. G. Buchanan B. Wilhelm G. Jones R. (2000). Biochemistry & Molecular biology of plants. Physiol. Mol. Biol. plants 1250–1318.

[B13] Dramé K. Clavel D. Repellin A. (2007). Water deficit induces variation in expression of stress-responsive genes in two peanut (Arachis hypogaea L.) cultivars with different tolerance to drought. Plant Physiol. Biochem. 45, 236–243. doi: 10.1016/j.plaphy.2007.02.002 17433701

[B14] Feller U. Crafts-Brandner S. J. Salvucci M. E. (1998). Moderately high temperatures inhibit ribulose-1,5-bisphosphate carboxylase/oxygenase (Rubisco) activase-mediated activation of rubisco. Plant Physiol. 116, 539–546. doi: 10.1104/pp.116.2.539 9490757 PMC35111

[B15] Furlan A. L. Bianucci E. Tordable M. del C. Castro S. Dietz K.-J. (2014). Antioxidant enzyme activities and gene expression patterns in peanut nodules during a drought and rehydration cycle. Funct. Plant Biol. 41, 704. doi: 10.1071/FP13311 32481025

[B16] Gangurde S. S. Kumar R. Pandey A. K. Burow M. Laza H. E. Nayak S. N.. (2019). “Climate-smart groundnuts for achieving high productivity and improved quality: Current status, challenges, and opportunities,” in Genomic Designing of Climate-Smart Oilseed Crops (Cham, Switzerland: Springer International Publishing), 133–172. doi: 10.1007/978-3-319-93536-2_3

[B17] Guy C. L. Huber J. L. Huber S. C. (1992). Sucrose phosphate synthase and sucrose accumulation at low temperature. Plant Physiol. 100, 502–508. doi: 10.1104/pp.100.1.502 16652990 PMC1075578

[B18] Halward T. Stalker H. Larue E. Kochert G. (1991). Genetic variation detectable with molecular markers among unadapted germ-plasm resources of cultivated peanut and related wild species. Genome 34, 1013–1020. doi: 10.1139/g91-156

[B19] Jain A. K. Basha S. M. Holbrook C. C. (2001). Identification of drought-responsive transcripts in peanut (Arachis hypogaea L.). Electron. J. Biotechnol. 4, 59–67. doi: 10.2225/vol4-issue2-fulltext-2

[B20] Jia J. Zhou J. Shi W. Cao X. Luo J. Polle A.. (2017). Comparative transcriptomic analysis reveals the roles of overlapping heat-/drought-responsive genes in poplars exposed to high temperature and drought. Sci. Rep. 7, 43215. doi: 10.1038/srep43215 28233854 PMC5324098

[B21] Kottapalli K. R. Rakwal R. Shibato J. Burow G. Tissue D. Burke J.. (2009). Physiology and proteomics of the water-deficit stress response in three contrasting peanut genotypes. Plant Cell Environ. 32, 380–407. doi: 10.1111/j.1365-3040.2009.01933.x 19143990

[B22] Laza H. E. Acosta-Martinez V. Cano A. Baker J. Mahan J. Gitz D.. (2023). Elevated [CO 2] enhances soil respiration and AMF abundance in a semiarid peanut agroecosystem. Agriculture, Ecosystems & Environment 355, 108592. doi: 10.1016/j.agee.2023.108592

[B23] Laza H. Baker J. T. Yates C. Mahan J. R. Burow M. D. Puppala N.. (2021). Effect of elevated CO2 on peanut performance in a semi-arid production region. Agric. For. Meteorol. 308–309, 108599. doi: 10.1016/J.AGRFORMET.2021.108599

[B24] Lohse M. Nagel A. Herter T. May P. Schroda M. Zrenner R.. (2014). Mercator: A fast and simple web server for genome scale functional annotation of plant sequence data. Plant Cell Environ. 37, 1250–1258. doi: 10.1111/pce.12231 24237261

[B25] Long S. P. Ainsworth E. A. Rogers A. Ort D. R. (2004). Rising Atmospheric Carbondioxide: plants FACE the future. Annu. Rev. Plant Biol. 55, 591–628. doi: 10.1146/annurev.arplant.55.031903.141610 15377233

[B26] Lu J. Pickersgill B. (1993). Isozyme variation and species relationships in peanut and its wild relatives (Arachis L. - Leguminosae). Theor. Appl. Genet. 85, 550–560. doi: 10.1007/BF00220913 24195929

[B27] Moore B. D. Cheng S. H. Sims D. Seemann J. R. (1999). The biochemical and molecular basis for photosynthetic acclimation to elevated atmospheric CO 2. Plant Cell Environ. 22, 567–582. doi: 10.1046/j.1365-3040.1999.00432.x

[B28] Noguchi K. Watanabe C. K. Terashima I. (2015). Effects of elevated atmospheric CO 2 on primary metabolite levels in arabidopsis thaliana col-0 leaves: an examination of metabolome data. Plant Cell Physiol. 56, 2069–2078. doi: 10.1093/pcp/pcv125 26423961

[B29] Novello N. Johnson J. B. Walsh K. B. Laza H. Naiker M. (2023). Potential Implications of Elevated CO2 on Physiochemical Parameters in Peanut (Arachis hypogaea L.) Genotypes, in: Foods 2023. MDPI Basel Switzerland, 26(1), 11. doi: 10.3390/Foods2023-15115

[B30] Parrott D. Yang L. Shama L. Fischer A. M. (2005). Senescence is accelerated, and several proteases are induced by carbon “feast” conditions in barley (Hordeum vulgare L.) leaves. Planta 222, 989–1000. doi: 10.1007/s00425-005-0042-x 16034594

[B31] Prasad P. V. V. Boote K. Allen L. Thomas J. (2003). Super-optimal temperatures are detrimental to peanut (Arachis hypogaea L.) reproductive processes and yield at both ambient and elevated carbon dioxide. Glob. Change Biol. 9, 1775–1787. doi: 10.1046/j.1529-8817.2003.00708.x

[B32] Pruthvi V. Rama N. Govind G. Nataraja K. N. (2013). Expression analysis of drought stress specific genes in Peanut (Arachis hypogaea, L.). Physiol. Mol. Biol. Plants 19, 277–281. doi: 10.1007/s12298-012-0156-0 24431496 PMC3656185

[B33] Qin H. Gu Q. Zhang J. Sun L. Kuppu S. Zhang Y.. (2011). Regulated expression of an isopentenyltransferase gene (IPT) in peanut significantly improves drought tolerance and increases yield under field conditions. Plant Cell Physiol. 52, 1904–1914. doi: 10.1093/pcp/pcr125 21920877

[B34] Sage R. F. Way D. A. Kubien D. S. (2008). Rubisco, Rubisco activase, and global climate change. J. Exp. Bot., 1581–1595. doi: 10.1093/jxb/ern053 18436544

[B35] Seijo G. Lavia G. I. Fernández A. Krapovickas A. Ducasse D. A. Bertioli D. J.. (2007). Genomic relationships between the cultivated peanut (Arachis hypogaea, Leguminosae) and its close relatives revealed by double GISH. Am. J. Bot. 94, 1963–1971. doi: 10.3732/ajb.94.12.1963 21636391

[B36] Sims D. A. Luo Y. Seemann J. R. (1998). Importance of leaf versus whole plant CO 2 environment for photosynthetic acclimation. Plant Cell Environ. 21, 1189–1196. doi: 10.1046/j.1365-3040.1998.00377.x

[B37] Vara Prasad P. V. Craufurd P. Q. Summerfield R. J. (1999). Fruit number in relation to pollen production and viability in groundnut exposed to short episodes of heat stress. Ann. Bot. 84, 381–386. doi: 10.1006/anbo.1999.0926

[B38] Vu J. C. V. Allen L. H. (2009). Growth at elevated CO2 delays the adverse effects of drought stress on leaf photosynthesis of the C4 sugarcane. J. Plant Physiol. 166, 107–116. doi: 10.1016/j.jplph.2008.02.009 18462832

[B39] Walker B. J. VanLoocke A. Bernacchi C. J. Ort D. R. (2016). The costs of photorespiration to food production now and in the future. Annu. Rev. Plant Biol. 67, 107–129. doi: 10.1146/annurev-arplant-043015-111709 26865340

[B40] Yamori W. von Caemmerer S. (2009). Effect of rubisco activase deficiency on the temperature response of CO2 assimilation rate and rubisco activation state: insights from transgenic tobacco with reduced amounts of rubisco activase. Plant Physiol. 151, 2073–2082. doi: 10.1104/pp.109.146514 19837817 PMC2786000

[B41] Yokoyama K. Ishijima S. A. Clowney L. Koike H. Aramaki H. Tanaka C.. (2006). Feast/famine regulatory proteins (FFRPs): Escherichia coli Lrp, AsnC and related archaeal transcription factors. FEMS Microbiol. Rev 30, 89–108. doi: 10.1111/j.1574-6976.2005.00005.x 16438681

[B42] Zinta G. AbdElgawad H. Domagalska M. A. Vergauwen L. Knapen D. Nijs I.. (2014). Physiological, biochemical, and genome-wide transcriptional analysis reveals that elevated CO _2_ mitigates the impact of combined heat wave and drought stress in *Arabidopsis thaliana* at multiple organizational levels. Glob. Change Biol. 20, 3670–3685. doi: 10.1111/gcb.12626 24802996

