# Supplementary material for: Peanut leaf transcriptomic dynamics reveals insights into the acclimation response to elevated carbon dioxide under semiarid conditions
Source: Front Plant Sci. 2025 Mar 27;15:1407574. doi: 10.3389/fpls.2024.1407574 (PMC11981908; doi:10.3389/fpls.2024.1407574)
Supplement: Supplementary Table 1 — List of genes and primers used for Real Time-Polymerase Chain Reaction (RT-PCR) gene expression validation of RNAseq transcriptomic in peanuts under elevated [CO2] and water stress. [file Table1.docx]

**Supplemental Table S1**. Primers used for QPCR validation of the effect of elevated CO_2_ on peanut leaf transcriptomic.
