# Supplementary material for: Peanut leaf transcriptomic dynamics reveals insights into the acclimation response to elevated carbon dioxide under semiarid conditions
Source: Front Plant Sci. 2025 Mar 27;15:1407574. doi: 10.3389/fpls.2024.1407574 (PMC11981908; doi:10.3389/fpls.2024.1407574)
Supplement: Supplementary Table 2 — Leaf RNAseq analysis summary-Photosynthesis. [file Table2.docx]

**Supplemental Table S2**. The effect of elevated CO_2_ on peanut leaf transcriptomic. Expression of photosynthesis (PS) related genes across the water stress episode (pre-water deficit [ pwd], and water deficit[wd].

| Treatment | Functional Group | Bincode | Name | log2F |  |
| --- | --- | --- | --- | --- | --- |
| pwd | Contig163582 | 1.1.1.2 | PS.lightreaction.photosystem II.PSII polypeptide subunits | 8.7 |  |
|  | Contig134551 | 1.2 | PS.photorespiration | -7.6 |  |
|  | Contig61770 | 1.2.2 | PS.photorespiration.glycolate oxydase | -5.5 |  |
| wd1 | Contig30942 | 1.1.1.1 | PS.lightreaction.photosystem II.LHC-II | 6.08 |  |
|  | Contig48516 | 1.1.1.1 | PS.lightreaction.photosystem II.LHC-II | 1.52 |  |
|  | Contig48530 | 1.1.1.1 | PS.lightreaction.photosystem II.LHC-II | 1.54 |  |
|  | Contig54247 | 1.1.1.1 | PS.lightreaction.photosystem II.LHC-II | 2.09 |  |
|  | Contig60415 | 1.1.1.1 | PS.lightreaction.photosystem II.LHC-II | 1.28 |  |
|  | Contig164921 | 1.1.1.1 | PS.lightreaction.photosystem II.LHC-II | 11.27 |  |
|  | Contig145537 | 1.1.1.2 | PS.lightreaction.photosystem II.PSII polypeptide subunits | 1.26 |  |
|  | Contig45806 | 1.1.2.1 | PS.lightreaction.photosystem I.LHC-I | 1.82 |  |
|  | Contig69587 | 1.1.2.2 | PS.lightreaction.photosystem I.PSI polypeptide subunits | 1.18 |  |
|  | Contig144433 | 1.1.2.2 | PS.lightreaction.photosystem I.PSI polypeptide subunits | 1.25 |  |
|  | Contig36381 | 1.1.5.2 | PS.lightreaction.other electron carrier (ox/red).ferredoxin | 1.59 |  |
|  | Contig184126 | 1.1.5.2 | PS.lightreaction.other electron carrier (ox/red).ferredoxin | 6.85 |  |
|  | Contig58431 | 1.2.6 | PS.photorespiration.hydroxypyruvate reductase | -5.67 |  |
|  | Contig134067 | 1.3.6 | PS.calvin cycle.aldolase | 1.32 |  |
|  | Contig132007 | 1.3.13 | PS.calvin cycle.rubisco interacting | -1.93 |  |
|  | Contig132010 | 1.3.13 | PS.calvin cycle.rubisco interacting | -1.95 |  |
|  | Contig132012 | 1.3.13 | PS.calvin cycle.rubisco interacting | -1.84 |  |
|  | Contig132013 | 1.3.13 | PS.calvin cycle.rubisco interacting | -1.96 |  |
|  | Contig132014 | 1.3.13 | PS.calvin cycle.rubisco interacting | -1.69 |  |
|  | Contig30942 | 1.1.1.1 | PS.lightreaction.photosystem II.LHC-II | -5.5 |  |
|  | Contig30944 | 1.1.1.1 | PS.lightreaction.photosystem II.LHC-II | -1.0 |  |
|  | Contig35853 | 1.1.1.2 | PS.lightreaction.photosystem II.PSII polypeptide subunits | -1.1 |  |
|  | Contig35854 | 1.1.1.2 | PS.lightreaction.photosystem II.PSII polypeptide subunits | -1.7 |  |
|  | Contig36124 | 1.1.1.2 | PS.lightreaction.photosystem II.PSII polypeptide subunits | -1.4 |  |
|  | Contig137737 | 1.1.1.2 | PS.lightreaction.photosystem II.PSII polypeptide subunits | 1.0 |  |
|  | Contig164633 | 1.1.1.2 | PS.lightreaction.photosystem II.PSII polypeptide subunits | 1.2 |  |
|  | Contig111892 | 1.1.2.2 | PS.lightreaction.photosystem I.PSI polypeptide subunits | -1.3 |  |
|  | Contig111893 | 1.1.2.2 | PS.lightreaction.photosystem I.PSI polypeptide subunits | -1.0 |  |
|  | Contig111896 | 1.1.2.2 | PS.lightreaction.photosystem I.PSI polypeptide subunits | -1.1 |  |
|  | Contig80941 | 1.2.5 | PS.photorespiration.serine hydroxymethyltransferase | -7.1 |  |
|  | Contig18365 | 1.2.6 | PS.photorespiration.hydroxypyruvate reductase | 1.7 |  |
|  | Contig58431 | 1.2.6 | PS.photorespiration.hydroxypyruvate reductase | 6.7 |  |
|  | Contig73114 | 1.3.4 | PS.calvin cycle.GAP | -1.0 |  |
|  | Contig73121 | 1.3.4 | PS.calvin cycle.GAP | -1.3 |  |
|  | Contig144099 | 1.3.7 | PS.calvin cycle.FBPase | -11.8 |  |
|  |  |  |  |  |  |
