# Supplementary material for: Peanut leaf transcriptomic dynamics reveals insights into the acclimation response to elevated carbon dioxide under semiarid conditions
Source: Front Plant Sci. 2025 Mar 27;15:1407574. doi: 10.3389/fpls.2024.1407574 (PMC11981908; doi:10.3389/fpls.2024.1407574)
Supplement: Supplementary Table 3 — Leaf RNAseq analysis summary-CHO. [file Table3.docx]

**Supplemental Table S3**. The effect of elevated CO_2_ on peanut leaf transcriptomic. Expression of carbohydrate metabolism (CHO) related genes across the water stress episode (pre-water deficit [pwd], water deficit[wd], and well-watered recovery [ww]

| Treatment | Functional Group | Bincode | Name | log2F |
| --- | --- | --- | --- | --- |
| pwd | Contig147757 | 2.1.2.1 | major CHO metabolism.synthesis.starch.AGPase | 1.5 |
|  | Contig127000 | 2.1.2.3 | major CHO metabolism.synthesis.starch.starch branching | -9.7 |
|  | Contig152514 | 2.1.2.3 | major CHO metabolism.synthesis.starch.starch branching | 1.5 |
|  | Contig152517 | 2.1.2.3 | major CHO metabolism.synthesis.starch.starch branching | 1.5 |
|  | Contig152522 | 2.1.2.3 | major CHO metabolism.synthesis.starch.starch branching | 2.9 |
|  | Contig13489 | 2.2.2.1.1 | major CHO metabolism.degradation.starch.starch cleavage.alpha amylase | 3.2 |
| wd1 | Contig63054 | 2.1.1.3 | major CHO metabolism.synthesis.sucrose.FBPase | 1.64 |
|  | Contig63056 | 2.1.1.3 | major CHO metabolism.synthesis.sucrose.FBPase | 1.48 |
|  | Contig63059 | 2.1.1.3 | major CHO metabolism.synthesis.sucrose.FBPase | 1.32 |
|  | Contig63066 | 2.1.1.3 | major CHO metabolism.synthesis.sucrose.FBPase | 1.78 |
|  | Contig63077 | 2.1.1.3 | major CHO metabolism.synthesis.sucrose.FBPase | 1.06 |
|  | Contig56831 | 2.1.2.1 | major CHO metabolism.synthesis.starch.AGPase | 1.00 |
|  | Contig147754 | 2.1.2.1 | major CHO metabolism.synthesis.starch.AGPase | 1.01 |
|  | Contig80655 | 2.1.2.2 | major CHO metabolism.synthesis.starch.starch synthase | 1.40 |
|  | Contig80659 | 2.1.2.2 | major CHO metabolism.synthesis.starch.starch synthase | 1.61 |
|  | Contig148320 | 2.1.2.4 | major CHO metabolism.synthesis.starch.debranching | 6.84 |
|  | Contig111014 | 2.1.2.60 | major CHO metabolism.synthesis.starch.ADP Glucose Phosphorylase | -1.05 |
|  | Contig54351 | 2.2.1.1 | major CHO metabolism.degradation.sucrose.fructokinase | 1.18 |
|  | Contig85385 | 2.2.1.1 | major CHO metabolism.degradation.sucrose.fructokinase | -1.44 |
|  | Contig81403 | 2.2.1.3.1 | major CHO metabolism.degradation.sucrose.invertases.neutral | 4.02 |
|  | Contig81473 | 2.2.1.3.1 | major CHO metabolism.degradation.sucrose.invertases.neutral | 4.02 |
|  | Contig84967 | 2.2.1.3.1 | major CHO metabolism.degradation.sucrose.invertases.neutral | -1.05 |
|  | Contig89856 | 2.2.1.3.3 | major CHO metabolism.degradation.sucrose.invertases.vacuolar | 1.43 |
|  | Contig89862 | 2.2.1.3.3 | major CHO metabolism.degradation.sucrose.invertases.vacuolar | 1.24 |

| Treatment | Functional Group | Bincode | Name | log2F |
| --- | --- | --- | --- | --- |
|  | Contig96005 | 2.2.1.3.3 | major CHO metabolism.degradation.sucrose.invertases.vacuolar | 2.80 |
|  | Contig129035 | 2.2.1.3.3 | major CHO metabolism.degradation.sucrose.invertases.vacuolar | 1.59 |
|  | Contig129403 | 2.2.1.5 | major CHO metabolism.degradation.sucrose.Susy | 1.57 |
|  | Contig80667 | 2.2.1.99 | major CHO metabolism.degradation.sucrose.misc | -6.04 |
|  | Contig60072 | 2.2.2.1.1 | major CHO metabolism.degradation.starch.starch cleavage.alpha amylase | -4.43 |
|  | Contig109494 | 2.2.2.1.1 | major CHO metabolism.degradation.starch.starch cleavage.alpha amylase | 4.34 |
|  | Contig158490 | 2.2.2.2 | major CHO metabolism.degradation.starch.starch phosphorylase | 1.73 |
|  | Contig82763 | 2.2.2.10 | major CHO metabolism.degradation.starch.laforin like phosphoglucan phosphatase (SEX4) | 7.47 |
| ww1 | contig137707 | 2.1.2.1 | major CHO metabolism.synthesis.starch.AGPase | 5.6 |
|  | contig96004 | 2.2.1.3.3 | major CHO metabolism.degradation.sucrose.invertases.vacuolar | 1.3 |
|  | contig13489 | 2.2.2.1.1 | major CHO metabolism.degradation.starch.starch cleavage.alpha amylase | 2.2 |
|  | contig39087 | 2.2.2.1.1 | major CHO metabolism.degradation.starch.starch cleavage.alpha amylase | 1.5 |
|  | contig27098 | 2.2.2.1.2 | major CHO metabolism.degradation.starch.starch cleavage.beta amylase | 3.4 |
|  | contig126706 | 2.2.2.2 | major CHO metabolism.degradation.starch.starch phosphorylase | 1.2 |
|  | contig75812 | 2.2.2.4 | major CHO metabolism.degradation.starch.D enzyme | 2.3 |
|  | contig126982 | 2.2.2.9 | major CHO metabolism.degradation.starch.limit dextrinase/ pullulanase | 1.1 |
