# Supplementary material for: Peanut leaf transcriptomic dynamics reveals insights into the acclimation response to elevated carbon dioxide under semiarid conditions
Source: Front Plant Sci. 2025 Mar 27;15:1407574. doi: 10.3389/fpls.2024.1407574 (PMC11981908; doi:10.3389/fpls.2024.1407574)
Supplement: Supplementary Table 4 — Leaf RNAseq analysis summary-Respiration. [file Table4.docx]

**Supplemental Table S4**. The effect of elevated CO_2_ on peanut leaf transcriptomic. Expression of respiratory-related genes across the water stress episode (pre-water deficit [pwd], water deficit[wd], and well-watered recovery [ww].

| Treatment | Functional Group | Bincode | Name | log2F |
| --- | --- | --- | --- | --- |
| pwd | Contig72075 | 5.3 | 'glycolysis.cytosolic branch.phosphofructokinase (PFK)' | 6.2 |
|  | Contig104297 | 5.3 | 'glycolysis.unclear/dually targeted.phosphoglycerate mutase' | 1.5 |
|  | Contig69242 | 5.1 | 'fermentation.ADH' | 2.3 |
|  | Contig48315 | 5.1 | 'fermentation.aldehyde dehydrogenase' | 6.1 |
|  | Contig67723 | 5.1 | 'fermentation.aldehyde dehydrogenase' | 1.2 |
|  | Contig59038 | 5.1 | 'TCA / org transformation.TCA.aconitase' | -7.8 |
|  | Contig142281 | 5.2 | 'mitochondrial electron transport / ATP synthesis.NADH-DH.type II.external' | 6.7 |
|  | Contig84300 | 5.2 | 'mitochondrial electron transport / ATP synthesis.alternative oxidase' | -1.3 |
| wd1 | Contig104778 | 6.4 | 'glycolysis.cytosolic branch.pyrophosphate-fructose-6-P phosphotransferase' | 4.67 |
|  | Contig104783 | 6.5 | 'glycolysis.cytosolic branch.pyrophosphate-fructose-6-P phosphotransferase' | 4.31 |
|  | Contig97535 | 4.1.11 | 'glycolysis.cytosolic branch.aldolase' | 2.05 |
|  | Contig84785 | 4.1.10 | 'glycolysis.cytosolic branch.phospho-enol-pyruvate carboxylase (PEPC)' | -1.96 |
|  | Contig72839 | 4.1.8 | 'glycolysis.unclear/dually targeted.phosphoglycerate mutase' | 5.40 |
|  | Contig108897 | 4.1.9 | 'fermentation.PDC' | 1.59 |
|  | Contig69242 | 4.1.15 | 'fermentation.ADH' | -1.06 |
|  | Contig50074 | 4.1.4 | 'fermentation.aldehyde dehydrogenase' | 5.43 |
|  | Contig59349 | 4.1.5 | 'fermentation.aldehyde dehydrogenase' | -1.10 |
|  | Contig12126 | 4.1.5 | 'gluconeogenesis / glyoxylate cycle.PEPCK' | 7.04 |
|  | Contig32834 | 4.1.5 | 'OPP.oxidative PP.6-phosphogluconolactonase' | 7.95 |
|  | Contig67363 | 4.3.10 | 'TCA / org transformation.TCA.pyruvate DH.E1' | 6.55 |
|  | Contig67366 | 4.3.12 | 'TCA / org transformation.TCA.pyruvate DH.E1' | -7.70 |
|  | Contig118942 | 4.3.12 | 'TCA / org transformation.TCA.malate DH' | 6.23 |
|  | Contig68779 | 4.3.12 | 'TCA / org transformation.other organic acid transformatons.malic' | -1.58 |
|  | Contig121524 | 9.4 | 'TCA / org transformation.other organic acid transformatons.malic' | 1.41 |

| Treatment | Functional Group | Bincode | Name | log2F |
| --- | --- | --- | --- | --- |
|  | Contig124519 |  | 'TCA / org transformation.other organic acid transformatons.atp-citrate lyase' | 1.94 |
|  | Contig107882 |  | 'TCA / org transformation.carbonic anhydrases' | 5.68 |
|  | Contig131903 |  | 'TCA / org transformation.carbonic anhydrases' | 1.19 |
|  | Contig131904 |  | 'TCA / org transformation.carbonic anhydrases' | 7.50 |
|  | Contig46259 |  | 'mitochondrial electron transport / ATP synthesis.NADH-DH.localisation not clear' | 7.75 |
|  | Contig76597 |  | 'mitochondrial electron transport / ATP synthesis.NADH-DH.localisation not clear' | 5.95 |
|  | Contig40669 |  | 'mitochondrial electron transport / ATP synthesis.cytochrome c' | 1.37 |
|  | Contig50961 |  | 'mitochondrial electron transport / ATP synthesis.uncoupling protein' | -1.00 |
| ww1 | Contig104783 | ww1 | glycolysis.cytosolic branch.pyrophosphate-fructose-6-P phosphotransferase | -5.1 |
|  | Contig118071 |  | glycolysis.cytosolic branch.glyceraldehyde 3-phosphate dehydrogenase (GAP-DH) | -1.4 |
|  | Contig154776 |  | glycolysis.cytosolic branch.non-phosphorylating glyceraldehyde 3-phosphate dehydrogenase (NPGAP-DH) | 1.0 |
|  | Contig112495 |  | glycolysis.cytosolic branch.3-phosphoglycerate kinase (PGK) | -1.1 |
|  | Contig22202 |  | glycolysis.unclear/dually targeted.aldolase | -5.0 |
|  | Contig104297 |  | glycolysis.unclear/dually targeted.phosphoglycerate mutase | 1.0 |
|  | Contig122278 |  | fermentation.PDC | 6.8 |
|  | Contig134375 |  | gluconeogenesis / glyoxylate cycle.pyruvate dikinase | 4.0 |
|  | Contig86390 |  | OPP.oxidative PP.6-phosphogluconolactonase | 4.5 |
|  | Contig104120 |  | OPP.oxidative PP.6-phosphogluconolactonase | 6.8 |
|  | Contig65908 |  | TCA / org transformation.other organic acid transformatons.IDH | 1.1 |
|  | Contig66033 |  | mitochondrial electron transport / ATP synthesis.NADH-DH.localisation not clear | -5.1 |
|  | Contig76597 |  | mitochondrial electron transport / ATP synthesis.NADH-DH.localisation not clear | 8.6 |
|  | Contig127456 |  | mitochondrial electron transport / ATP synthesis.NADH-DH.localisation not clear | -1.5 |
|  | Contig61079 |  | mitochondrial electron transport / ATP synthesis.electron transfer flavoprotein | 5.1 |
|  | Contig45648 |  | mitochondrial electron transport / ATP synthesis.alternative oxidase | 5.7 |
|  | Contig127030 |  | mitochondrial electron transport / ATP synthesis.cytochrome c oxidase | 1.1 |
|  | Contig117977 |  | mitochondrial electron transport / ATP synthesis.F1-ATPase | -6.2 |
